# Examining the knowledge, attitudes and practices of domestic and international university students towards seasonal and pandemic influenza

**DOI:** 10.1186/1471-2458-12-307

**Published:** 2012-04-26

**Authors:** Holly Seale, Jackie PI Mak, Husna Razee, C Raina MacIntyre

**Affiliations:** 1 School of Public Health and Community Medicine, Faculty of Medicine, The University of New South Wales, South Wales, Australia; 2 Faculty of Medicine, The University of New South Wales, South Wales, Australia; 3 National Centre for Immunisation Research and Surveillance of Vaccine Preventable Diseases (NCIRS), The Children’s Hospital at Westmead and Discipline of Paediatrics and Child Health, University of Sydney, New South Wales, South Wales, Australia; 4 School of Public Health & Community Medicine, Level 3, Samuels Building, Faculty of Medicine, University of New South Wales, Sydney, 2052, Australia

## Abstract

**Background:**

Prior to the availability of the specific pandemic vaccine, strategies to mitigate the impact of the disease typically involved antiviral treatment and “non-pharmaceutical” community interventions. However, compliance with these strategies is linked to risk perceptions, perceived severity and perceived effectiveness of the strategies. In 2010, we undertook a study to examine the knowledge, attitudes, risk perceptions, practices and barriers towards influenza and infection control strategies amongst domestic and international university students.

**Methods:**

A study using qualitative methods that incorporated 20 semi-structured interviews was undertaken with domestic and international undergraduate and postgraduate university students based at one university in Sydney, Australia. Participants were invited to discuss their perceptions of influenza (seasonal vs. pandemic) in terms of perceived severity and impact, and attitudes towards infection control measures including hand-washing and the use of social distancing, isolation or cough etiquette.

**Results:**

While participants were generally knowledgeable about influenza transmission, they were unable to accurately define what ‘pandemic influenza’ meant. While avian flu or SARS were mistaken as examples of past pandemics, almost all participants were able to associate the recent “swine flu” situation as an example of a pandemic event. Not surprisingly, it was uncommon for participants to identify university students as being at risk of catching pandemic influenza. Amongst those interviewed, it was felt that ‘students’ were capable of fighting off any illness. The participant’s nominated hand washing as the most feasible and acceptable compared with social distancing and mask use.

**Conclusions:**

Given the high levels of interaction that occurs in a university setting, it is really important that students are informed about disease transmission and about risk of infection. It may be necessary to emphasize that pandemic influenza could pose a real threat to them, that it is important to protect oneself from infection and that infection control measures can be effective.

## Background

Public cooperation in complying with infection control measures is required to minimize the spread of infectious diseases. Previous studies have demonstrated the positive correlation between willingness to adhere to the recommendations around infection control practices and perceived infectiousness and severity of the disease [1-4], perceptions about the effectiveness of control measures [5] and trust in the information being provided by national and international public health authorities [1].

From the literature published to date on the general public’s risk perceptions and behaviour changes during the 2009 influenza A/H1N1 pandemic [1,6,7], higher risk perception scores were reported from Asian countries than from Western countries. For example, participants from studies conducted in India [8], Saudi Arabia [6] and Hong Kong [9] expressed higher concern and perceived susceptibility levels than the respondents from studies conducted in the UK [1] and Australia [3]. While these variations may be correlated with methodological issues or the time period during the 2009 pandemic in which the study was conducted (i.e. May 2009 versus November/December 2009), if the trends are accurate it has the potential to affect the speed and extent to which infection control measures are accepted.

During the height of the pandemic, we undertook a study which aimed to measure the perceptions and responses of staff and students at our University [10]. While a large proportion of the sample reported either “no anxiety” or “disinterest”, Asian respondents were significantly (p < 0.001) more likely to believe that the pandemic was serious compared to their counterparts from other regions. Although, most participants reported not adopting any specific behaviour changes, those who did were significantly more likely to be of Asian origin.

In order to further explore these trends amongst our domestic and international university students, we used qualitative methods to explore their attitudes, risk perceptions and adoption of health behaviour interventions against seasonal and pandemic influenza.

## Methods

### Study design

This study was carried out from May to August 2010. Qualitative semi-structured interviews were undertaken at the University of New South Wales (UNSW) in Sydney, Australia. The relevant Human Research Ethics Committee located at the university approved this study.

### Participants

Students attending the main campus of the university were approached to participate in the study. Two methods were used to identify potential participants. Firstly, the interviewer (JP) directly approached a convenience sample of students who were located in the food halls and recreation areas of the university campus and invited them to participate. In the latter half of the study, a snowball approach was used. The snowball approach was adopted due to problems with identifying and recruiting postgraduate students. They constitute a considerably smaller percentage of the total student body, often are enrolled externally and attend classes in the late afternoon/evening. Students were classified on the basis of their enrolment status: undergraduate vs. postgraduate, and domestic vs. international. We aimed to recruit a sample of students from each classification and hence we firstly screened the student to identify their enrolment status. Students enrolled in the bachelor of medicine were excluded as it was assumed they would not be representative of the general student body, and would have had more exposure to issues surrounding disease spread and control. Participation was voluntary and written consent was obtained. During the study period, pandemic influenza H1N1 activity remained low and sporadic cases of pandemic influenza continued to be reported without evidence of sustained community transmission [11].

### Data collection

The study researchers collaboratively developed an interview guide. Questions were shaped to cover the key areas of interest that included: knowledge, perceived severity, risk perceptions and concerns towards seasonal and pandemic influenza and personal health seeking behaviours and practices. Small variations in the questions were used to provide relevance for the overseas students. For example, we explored whether the international students believed their personal risk varied when located at home versus while residing in Australia and whether they had adopted or discontinued any health related behaviours whilst studying in Australia. We were not prescriptive around the term ‘pandemic influenza’; instead we left it up to the student to interpret what they felt it meant to them. Pre-designed prompts were employed throughout the interview to trigger interviewees’ thought. An interview face sheet was used to collect demographic information (sex, age, enrolment status etc.) from the participants. All interviews were conducted by JM and lasted up to one hour in length.

### Data analysis

The interviews were recorded and transcribed verbatim. Two investigators (HS and JM) developed a list of themes after the analysis of one-quarter of the transcripts. An agreed framework was then applied to another subsample of transcripts and further modified. Using this final framework, all of the transcripts were analysed and coded. Text was organized within the identified themes of the developed framework. No software was used in the process.

## Results

A total of 20 university students’ aged ≥18 years completed the interview (RR: 35 %). The participants ranged in age between 21 to 30 years and 70 % were born overseas (14/20). International students were over-represented in our sample (50 %, 10/20) compared to the actual proportion enrolled at UNSW (25 %).

There was a reasonably level of knowledge amongst the participants about the transmission modes and common symptoms of influenza and the common cold. A number of international students associated the occurrence of seasonal influenza with the temperature drop in winter; however they did not elaborated on the mechanisms of the connection.

“....people tend to get sick, the flu during winter because of the change in temperature, it gets colder….” (International postgraduate student)

Many of the participants were unable to accurately define what pandemic influenza was. While avian flu or SARS were mistaken as examples of past pandemics, almost all participants were able to associate the recent “swine flu” situation as an example of a pandemic event. In comparison to seasonal influenza and colds, participants generally perceived pandemic influenza as being more serious. Pandemic influenza was associated with increased numbers of medical consultations/hospitalizations and a higher mortality rate. However, there were a few sceptical participants who were doubtful about the actual disease impact and felt that it was only “promoted” as being serious by the government and the media.

Young children and the elderly were nominated as being the most vulnerable groups during a pandemic outbreak due to their ‘sub-optimal immune systems’. Participants believed that children were less conscious about hygiene and were therefore more likely to be exposed to other infected children or contaminated objects in a school environment. On the other hand, teenagers and young adults (20s-30s), the ‘physically and socially healthy’ and the ‘well educated’, were considered to be at lowest risk of contracting pandemic influenza.

In regards to differences in risk between racially or culturally diverse groups, one participant commented that people or cultures that have frequent proximate interaction with each other were at risk of contracting the disease during pandemic. While another suggested that in countries where there are higher levels of respect for traditional medicines over western medicines, people may also be at risk. Amongst the international students it was suggested that the risk of contracting pandemic influenza is higher for people in their ‘home towns’ because of differences in their health care system, population density, personal hygiene practices and environmental quality.

“…culture…..where they interact with a lot of people… such as Italians, they’re very outgoing…, lots of interaction, proximate to each other, perhaps they’re more prone,” (Domestic postgraduate student)

“….some cultures where they have let say more respect for traditional medicine than modern medicine…….are also going to be a problem…that’s why lots of pandemic in say Asia and Africa, and not so much in Europe or America” (International postgraduate student)

Not surprisingly, only a few postgraduate participants stated that university students were at risk of catching pandemic influenza. When participants were required to rate their self-perceived risk of contracting pandemic influenza in Australia during an outbreak, almost all of them rated themselves at the low end of the scale. Being young and leading a healthy lifestyle were the major reasons provided to justify the low self-perceived risks levels. Only two overseas participants considered themselves at the ‘relatively high risk’ end. However, they presented very different justifications for this ranking, as one thought that their ‘adventurous lifestyle’ put them at risk and the other because of the dynamic nature of the university environment.

“…may be I travel a lot more than the other people, and I go to polluted environments, institutions…. and I meet people I don’t know. “(International undergraduate student)

Five of the international students perceived themselves to be at a higher risk of catching pandemic influenza in their ‘home town’ than in Australia. Differences in population density, quality of transport, connectivity to other countries, hygiene levels, accessibility to and quality of health care were the main reasons given for the differences.

Regular hand washing, cough etiquette (covering mouth and nose when coughing or sneezing), and avoiding the sick were suggested as good strategies to prevent becoming infected with pandemic influenza. The use of social distancing/isolation or masks/respirators was not very popular. Social avoidance was considered to be the most difficult intervention to comply with and impractical due to the vast amount of human interaction existing in the society. One international student also felt that it was impolite to maintain a distance to a sick acquaintance or relative. The use of masks was dismissed, as they were considered uncomfortable, inconvenient and unnecessary. Moreover, participants believed that wearing mask would cause embarrassment and social stigma.

“…is he ill or is he dangerous something like that? …like the old leprosy people in Europe…” (Domestic postgraduate student)

“…people would look at you weird here” (International undergraduate student)

## Discussion

Outbreaks of seasonal influenza amongst student and university populations have been previously reported [12,13]. These outbreaks have resulted in increased absenteeism, impaired school performance, and increased health care utilization [14]. The first reported university outbreak of 2009 pandemic H1N1 occurred at the University of Delaware (UD), affecting an estimated 10 % of the student population. It spread rapidly through the University of Delaware community with a surge in illness over a 2-week period. Although severe illness was rare in this instance, the authors documented that the outbreak caused a substantial burden and challenge to the university health care system [15]. In Japan, Uchida et al. reported that the infection rate among university students they surveyed ranged from 4.3 % to 15.5 % during the 2009 pandemic [16]. The authors suggested that continued exposure to sick individuals and disease transmission occurred during the pandemic, mainly through university club activities.

While our participants were knowledgeable about the modes of transmission of influenza, very few were able to accurately describe what ‘pandemic influenza’ actually meant. The participants had heard of ‘swine flu’, however only a few demonstrated a high level of knowledge around how it originated. There was a lot of confusion around the role that animals play in regard to ‘pandemics’. Unconfirmed beliefs and misconceptions regarding pandemic influenza H1N1 2009 have been previously documented [17,18].

In accordance with most of the pre- and post pandemic general public studies conducted worldwide [1,2,8,19,20], our participants held a common belief that they were not at risk of acquiring the disease. Amongst our participants, it was felt that they were protected against the infection because they were ‘fit and young’. This sense of non-vulnerability has also been previously documented in our previous university study [10] and amongst dormitory housed university students (aged18-23 years) in the USA [21]. However, given the low level of comprehension about pandemic influenza, the general public may be over or underestimating their level of risk towards acquiring the disease and the health consequences if infected (serious illness, need for hospitalization, mortality risk).

As highlighted through the interviews, our students believed in the classic picture of morbidity attributable to the flu, such that only the very young, the elderly, those with co-morbidities and those with weakened immunity are at risk. This result is consistent with the risk groups identified by participants in previous studies [4]. Given this low level of anxiety towards the pandemic, it is perhaps not surprising that the students did not undertake any behavioural changes in response to the H1N1 pandemic, as highlighted here and in our previous study [10].

During the 2009 H1N1 pandemic, posters developed by the Commonwealth Department of Health and Ageing and UNSW were placed in high traffic areas. They focused on: (1) encouraging faculty, staff and students to stay at home if symptomatic (i.e. with a fever, cough, and runny nose) and to protect each other; (2) cough/sneeze etiquette (i.e. “cover your mouth and nose when you cough and sneeze” and “dispose of used tissues in the bin) (3) hand hygiene (i.e. “Wash your hands properly and regularly”). Our participants considered regular hand washing, cough etiquette (covering mouth and nose when coughing or sneezing), and avoiding the sick as good strategies to prevent infection. During the early and peak pandemic periods, hand washing was found to be the most accepted intervention among university students in Hong Kong [22], Korea [23], United States [24] and Australia [10]. Young people such as our university students may be more amenable to hand hygiene as a strategy because of a number of reasons. Firstly, these practices are community learnt and represent actions that the person has been encouraged to carry out from a young age. Secondly, hygiene-based measures pose minimal disruptions to daily routine. However, this is just a hypothesis and was not explored in depth in the study.

Amongst our participants, mask use, as an infection control strategy was extremely unpopular. In many western settings, where medical mask/respirator use is generally restricted to the hospital setting, it is not unanticipated that people would associate embarrassment and social stigma with the use of these products. At the University, it is extremely rare to see a student wearing a medical mask. This maybe because students believe that masks are uncomfortable, inconvenient and unnecessary. Habit is an important influence on routine behaviour [25], including hygiene behaviour [26], such that despite their best intentions people may find it difficult to implement new hygiene measures during a pandemic if they have not previously made these a habit.

The implementation of infection control behaviours appears to depend on a number of environmental (e.g. time, energy, availability of facilities, social norms), and motivational (e.g. social responsibility, social relationships, selfishness) factors. In the future however, the level of adoption of measures such as masks will fluctuate with changes in perceptions of risks and the perceived infectiousness and severity of the disease.

The use of voluntary home quarantine, social distancing, and school dismissal to prevent the transmission of pandemic influenza is a standard inclusion in most countries pandemic plans [27-29]. However, lower acceptance of isolation and social distancing, which can disrupt routine and enjoyable activities, has been observed in prior studies [30,31]. When participants were asked to comment on how they felt about the use of these interventions they stated that they were not in favour of adopting these actions and would find them extremely difficult to comply with. During an outbreak of pandemic H1N1 virus infection at a large public university in April 2009, Mitchell et al. undertook an online survey of students, faculty, and staff to assess knowledge of and adherence to university recommended non-pharmaceutical interventions [24]. They found that amongst the students with an acute respiratory infection (ARI), 44 % reported leaving campus for >1 day while sick, 35 % had visitors and only 34 % reported missing days of class. Most students attended class or work, went out in public, and participated in purely social activities (including having visitors) while having an ARI. Aside from not wanting to miss these important events, it could be suggested that low risk perceptions and mixed messages about the severity of the 2009 influenza H1N1 pandemic and about the actual need for isolation and social distancing would probably have contributed to a low acceptance rate.

There are a number of logistical issues that universities and other institutions need to contend with instigating measures such as isolation. For example, universities may have large numbers of students living on or around the campus. While some of these facilities are self-contained, others have large common dining, entertainment and study rooms. The difficulty of introducing home quarantine in this setting is that many of these students (especially international and interstate students) may be unable to leave the campus facilities and would end up having to care and cater for themselves. Given the inevitability of future disease outbreaks or pandemic, universities must undertake efforts ensure that the needs of the students are catered for in these situations. Students, their parents, and other members of the university community must be involved with planning for these events so that feasible action plans are developed. These plans must ensure that there is continuity for the student.

A strength of this study was using interviews that allowed to uncover in greater depth the attitudes and perceptions of the students. However, there are several limitations in this study. These include: (1) over reporting: as the study was conducted through face-to-face interviews with our interviewer, it may have resulted in an over-reporting of infection control behaviours to avoid embarrassment or judgement; (2) recall bias: some questions in the interview guide required participants to recall their past experiences during the 2009 H1N1 pandemic, therefore recalling errors may have occurred; (3) representation: as the study was undertaken one year after the pandemic, the responses received may not represent the attitudes that participants held during the pandemic; (4) participation rate was low.

Communicating to students effectively about the spread of influenza and the need to adopt preventative measures on a large campus presents a challenge. University officers need to find a balance between promoting and educating, while trying not to incite unnecessary fear. In the event of prolonged public health threats, such as infectious disease disasters, online messaging and regularly updated web sites have been shown to be timely and effective in providing risk communication and health messages [32]. However, it has also been demonstrated that pandemic influenza-specific web sites are among the least accessible and most difficult to understand compared with web sites addressing other types of disasters [33]. Poor accessibility can significantly undermine the effectiveness of university pandemic preparedness efforts and limit the ability of individuals to make well informed decisions during pandemics [34]. Other mediums favoured by young adults such as popular internet sites (Facebook or twitter) should be considered as a possible means of information provision to this susceptible cohort and in increasing uptake of preventative health advice. Education campaigns targeting young adults could also utilise the university networks and information gateways, or distribute information through university-wide emails and newsletters.

## Conclusions

Given the high levels of interaction that occurs in a university setting, it is really important that students are informed about disease transmission and about risk of infection. It may be necessary to emphasize that pandemic influenza could pose a real threat to them, that it is important to protect oneself from infection and that infection control measures can be effective. Our participants believed that it would be extremely difficult to comply with infection control measures such as social distancing. In this university setting, practical measures may also be needed to support implementation, such as education, reminders and provision of hand gel.

## Competing interests

Raina MacIntyre receives funding from influenza vaccine manufacturers GSK and CSL Biotherapies for investigator-driven research. These payments were not associated with this study. The remaining authors have no competing interests.

## Authors’ contributions

HS/JM participated in the design of the study and interview guide, undertook the interview and transcription, performed the analysis and drafted the manuscript. CRM/HR assisted with the analysis and reviewed the manuscript. All authors read and approved the final manuscript.

## Pre-publication history

The pre-publication history for this paper can be accessed here:

http://www.biomedcentral.com/1471-2458/12/307/prepub
